# Labor conditions and the meanings of nursing work in Barcelona

**DOI:** 10.1590/1518-8345.2342.2947

**Published:** 2018-06-18

**Authors:** Alberto Granero, Josep M Blanch, Paola Ochoa

**Affiliations:** 1 PhD, Researcher, Corporación Sanitaria Parc Taulí -1, Hospital Universitario, Sabadell- Barcelona, Catalonia, Spain. Member of PETRO (Personas que Trabajan em Organizaciones, People Working in Organizations) research group.; 2 PhD, Full Professor, Autonomous University of Barcelona (UAB), Psychology, Barcelona, Catalonia, Spain. San Buenaventura University (USB), Psychology, Cali, Colombia. Director of PETRO and WONPUM (Working Under New Public Management) research groups.; 3 PhD, Professor, Politécnica del Litoral High School, ESPOL-ESPAE, Graduate School of Management, Campus Peñas Malecón 100 y Loja, P.O., Guayaquil, Box 09-01-5863, Ecuador. Member of PETRO and WONPUM research groups.

**Keywords:** Nursing, Work, Occupational Health, Working Conditions, Workload, Quality of Life

## Abstract

**Objective:**

to analyze the relationship between the quantitative assessment of working conditions and the qualitative perception of one’s own work experience.

**Method:**

a sample of 1,760 nursing professionals from Barcelona answered a questionnaire assessing their working conditions and summarized their own current work experience in five key words.

**Results:**

the textual corpus of the meanings of nursing work included 8043 lexical forms, which were categorized and codified. Respondents who rated their work conditions the highest expressed a vision of their work in terms of autonomy, achievement and well-being, while those who rated their work conditions the lowest talked mostly of exhaustion, depersonalization and negative climate. A correspondence analysis showed a close relationship between the quantitative assessments of working conditions and the verbal codes of the meaning of work.

**Conclusions:**

the meanings given to work were not only consistent with the numerical evaluations of the working conditions but also made them more understandable. The information obtained poses challenges for reflection and indicates ways to promote the positive aspects and prevent the negative conditions of nursing work.

## Introduction

The working conditions of health professionals, and particularly those of nursing professionals, have undergone profound changes in light of the general changes in the world of work. The scientific literature has analyzed the impact of working conditions on well-being and professional performance, as well as their multiple side effects on occupational health and the quality of the service provided. These effects appear as a negative spiral of care pressure, staff shortage, task overload, time deficit to execute everything and to do so well, distress and burnout, absenteeism and presentism, rotation and abandonment of the workplace and profession^1^ ^-^ ^11^. The role of moderating variables in some of these effects, such as control of process and work content, horizontal and vertical social support, degree of adjustment between demands and labor resources, and the work-family balance or emotional load, has also been studied^12^ ^-^ ^15^. Some research reports the economic and human cost of the lack of prevention of psychosocial risks in work in general and particularly in health care services^16^ ^-^ ^18^.

Another important topic of contemporary research in this field is the dynamics by which people give meaning and significance to their work in different sociocultural and organizational contexts and the role these cognitive processes play not only in shaping work experience but also in how professional practice is carried out^19^ ^-^ ^23^. In this regard, psycho-sociological theories about *the meaning of working* argue that the meanings that people ascribe to their work do not derive only from immediate situations, contexts and conjunctures but also from a complex construction process involving values, ideals, goals, norms, rhetoric, strategies, beliefs, aspirations and sociocultural and personal expectations about work, profession and career. 

In recent decades, the development of the nursing profession has followed a paradoxical development at both the global and local levels: on the one hand, training in skills and material and technological resources for professional performance has improved, while on the other, the conditions of work have become more hard, complex and difficult. This is precisely what happened in the environment of Barcelona, ​​where, over the last half-century, the profession went from basic training as a *Technical Medical Assistant* (1953-1979), through the *Diploma in Nursing* (1977-2012) to the *Graduate degree in Nursing* (begun in 2009), which opened the way for the second- and third-cycle training (Master’s and Doctorate) of nursing as an autonomous discipline and the development of the profession in the areas of assistance, teaching, research and management^24^. In contrast to this positive trend, the conditions of application of this work potential evolved in the opposite direction in a series of organizational aspects. The initial situation was marked by a high ratio of patients per professional, well above the European average, which already entails a high care pressure. On this basis, a new organization and business management of health care work was implemented in recent decades, reinforced by certain policies to cope with the recent economic crisis. In this context, the imposition of measures to reduce the public deficit, especially the health budget, entailed reduced staffing levels and increased annual work time, posing obstacles to professionals’ careers and increasing psychosocial risks associated with burnout. This contemporary tension between the improvement of the professional qualifications and the worsening of the practice conditions of the profession has induced nursing professionals to reflect on the value, meaning and usefulness of their work. In this scenario, the objective of this research was to analyze the relationship between the quantitative assessment of working conditions and the qualitative perception of one’s own work experience in nursing professionals.

## Method

Through the website of the Official College of Nurses of Barcelona (COIB) and some social networks of sector associations, 1,760 people, from a finite population of 32,463 nursing professionals, were accessed through simple random sampling. These individuals were working nurses in the province of Barcelona in June 2014. The size of this sample provided a confidence level of 99% and a confidence interval of 3%. The characteristic profile of the participants (which reproduced with remarkable precision that of the reference population) was that of women (85.9%), 41.9 years old (standard deviation SD = 10.4), with 18.3 years (SD = 10.8) of seniority since graduation and 13.5 years (SD= 10.0) in the workplace, with a general education (95.5%), stable or permanent contract (73.0%), full-time employment (76.1%), day shift (80.5%), in the health care industry (81.0%), hospital or clinic (53.3%), public (32.7%) or mixed ownership (57.7%). All of them voluntarily answered a survey that collected information on the central variables of the study by including the *Questionnaire on Working Conditions* ^25^ ^)^ (*Cuestionario de Condiciones de Trabajo*, cCT), composed of sets of closed items with a Likert-type format with ranges from 0 to 10 and an open question regarding the meaning of the work that invited respondents to summarize the actual work experience itself in 5 “keywords.” The lexical forms obtained from the answers were later categorized and codified. Finally, the respondents filled out a section of sociodemographic and work data.

**Figure 1:**
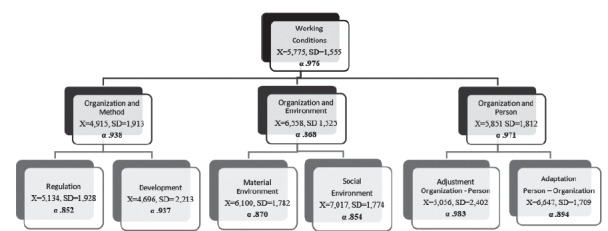
Theoretical Model of cCT: Cronbach’s Alpha (α), Mean (X) and Standard Deviation (SD) of the Questionnaire, Factors and Scales, Barcelona, CA, Spain, 2014-2015

The cCT is based on a theoretical model according to which the working conditions are structured around a triple relationship of the organization with the method, the environment and the person. The questionnaire evaluates the psychosocial components of working conditions (WC) that, depending on their form of presence and level of intensity, can function as protectors and promoters of health, well-being and quality of work life or as psychosocial risk factors. The cCT includes 44 closed items, presented in a Likert-type format of 11 ranks ranging from 0 (minimum value) to 10 (maximum value). The obtained psychometric data adequately reproduced the proposed theoretical model structure and showed a high internal consistency, with alpha values ​​ranging from 0.852 to 0.983. In *figure 1*, the mean, standard deviation and Cronbach’s alpha of the entire questionnaire are specified, and each of the factors and scales are included.

The mixed design of the research combined the qualitative technique of the analysis of textual content for the study of meanings expressed by keywords and the quantitative techniques of descriptive statistical analysis of the WCs and of factorial analysis of correspondences between WC and meaning of work (MW). For the correspondence analysis, the WC-related scores were divided into four categories: “very bad” WC (X = 0-2.5), “bad” WC (X = 2.5-5), “good” WC (X = 5- 7.5) and “very good” WC (X = 7.5-10). In the descriptive statistical analysis, the quantitative variables were presented in terms of mean (X) and standard deviation (SD), while the qualitative variables were in the form of frequencies and percentages. In the factorial analysis of correspondences, the chi square (χ²) was used to determine the relationships between the WC and MW variables from the data in the contingency table.

The project of this research was approved by the Bioethics Commission of the University of Barcelona. In its development, the international rules regarding informed consent, confidentiality of data of participants and institutions, safeguarding the anonymity of the answers, commitment of return of results and scientific and responsible use of the information were applied. 

## Results

All the lexical forms were categorized and codified according to a dictionary constructed from a theoretical model according to which the meanings of the work are distributed along a bipolar semantic continuum. One of these slopes contains negative connotations of the work experience, such as malaise and dissatisfaction, as well as various aspects covered by the model of *burnout,* such as physical and emotional exhaustion, depersonalization, cynicism and inefficacy. On the other side are positive work meanings associated with well-being and satisfaction, realization and efficacy, as well as the components of the *work engagement* model (vigor, dedication and absorption)^21^ ^-^ ^23^.

The mean valuation of WC, in scales ranging from 0-10, was 5.77 (SD=1.555), as shown in Figure 1. On the other hand, the textual corpus of the MW included 8043 lexical forms that, in turn, were found to be of the same order of magnitude; 62% had positive connotations (care, companionship, commitment, etc.) and the remaining 38% had negative connotations (exhaustion, malaise, overload, etc.).

A correspondence analysis showed a close and statistically significant relationship between the quantitative assessments of working conditions and the verbal codes of meaning of the work (χ^2^ = 1434.01, p <0.001 and degrees of freedom = 93), as shown in Table 1.

**Table 1:** Correspondence analysis between evaluation levels of working conditions and codes of meaning of work. Barcelona, CA, Spain, 2014-2015.

Meaning of Work	Mass	Score in dimension		Inertia	Contribution				
					From the points to the inertia of the dimension		From the dimension to the inertia of the point		
		1	2		1	2	1	2	Total
WC Very Bad	0,027	-1,340	-1,521	0,026	0,122	0,715	0,754	0,213	0,967
WC Bad	0,280	-0,843	0,257	0,082	0,491	0,208	0,978	0,020	0,998
WC Good	0,575	0,273	-0,084	0,019	0,106	0,046	0,892	0,018	0,911
WC Very Good	0,118	0,984	0,154	0,050	0,281	0,031	0,923	0,005	0,927
Total	1,000			0,178	1,000	1,000			
Overload	0,057	-0,268	-0,199	0,002	0,010	0,025	0,784	0,095	0,879
Bad management	0,014	-1,214	-0,237	0,009	0,051	0,009	0,974	0,008	0,982
Disorganization tasks	0,012	-0,887	0,583	0,004	0,022	0,044	0,905	0,086	0,991
Negative Climate	0,031	-0,798	0,462	0,009	0,049	0,074	0,930	0,068	0,998
Injustice	0,014	-1,187	0,513	0,009	0,050	0,042	0,958	0,039	0,997
Inappropriate work	0,015	-0,585	0,439	0,002	0,013	0,033	0,887	0,109	0,996
Lack of Resources	0,004	-0,763	-0,819	0,001	0,006	0,030	0,787	0,199	0,986
Poor socioeconomic Conditions	0,063	-0,523	-0,397	0,008	0,043	0,112	0,853	0,108	0,961
Dissatisfaction	0,017	-1,214	0,002	0,011	0,063	0,000	0,978	0,000	0,978
Discomfort	0,060	-0,989	-0,361	0,025	0,144	0,088	0,960	0,028	0,988
Exhaustion	0,070	-0,499	0,106	0,008	0,043	0,009	0,912	0,009	0,921
Organizational Cynicism	0,013	-1,185	0,293	0,008	0,045	0,013	0,959	0,013	0,972
Depersonalization	0,005	-1,538	-1,665	0,007	0,029	0,155	0,722	0,186	0,908
Inefficacy	0,006	-1,425	1,624	0,006	0,029	0,173	0,762	0,217	0,979
Good socioeconomic Conditions	0,019	0,498	0,021	0,002	0,012	0,000	0,941	0,000	0,942
Opportunities	0,043	0,593	0,000	0,006	0,037	0,000	1,000	0,000	1,000
Satisfaction	0,035	0,597	0,064	0,006	0,031	0,002	0,894	0,002	0,896
Wellbeing	0,049	0,690	0,033	0,010	0,057	0,001	0,973	0,000	0,974
Vigor	0,037	0,559	0,146	0,005	0,028	0,009	0,917	0,014	0,931
Commitment	0,052	0,580	0,040	0,007	0,044	0,001	0,985	0,001	0,986
Ethics	0,017	0,303	0,046	0,001	0,004	0,000	0,728	0,004	0,732
Good social Relations	0,055	0,450	0,040	0,005	0,028	0,001	0,979	0,002	0,980
Realization	0,014	0,789	-0,052	0,004	0,021	0,000	0,999	0,001	1,000
Efficacy	0,069	0,523	-0,057	0,008	0,046	0,002	0,992	0,003	0,994
Competences	0,009	0,303	-0,046	0,001	0,002	0,000	0,640	0,003	0,643
Care	0,135	0,274	-0,104	0,004	0,025	0,016	0,925	0,029	0,955
Responsibility	0,045	0,397	0,045	0,003	0,017	0,001	0,887	0,003	0,890
Autonomy	0,012	0,875	0,173	0,004	0,023	0,004	0,883	0,008	0,891
Little Recognition	0,014	-0,868	0,863	0,005	0,026	0,118	0,803	0,174	0,977
Recognition	0,007	0,277	0,668	0,001	0,001	0,036	0,308	0,392	0,700
Economic Recognition	0,008	0,069	0,044	0,000	0,000	0,000	0,127	0,011	0,138
Total	1,000			0,178	1,000	1,000			

**Figure 2:**
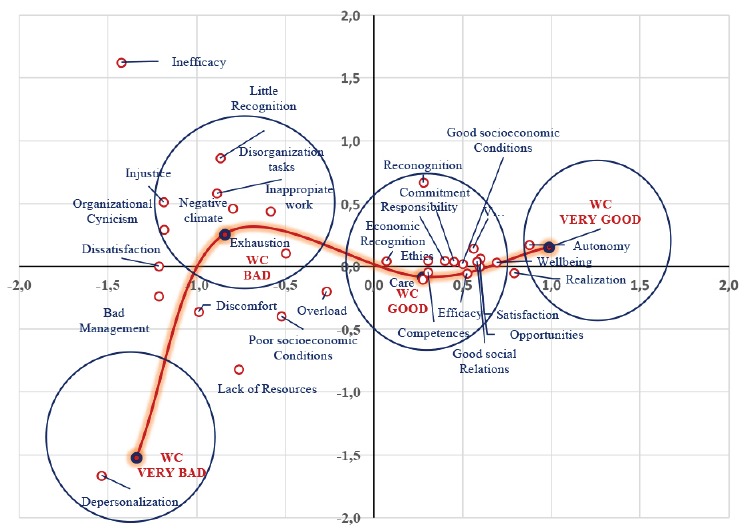
Conditions and Meanings of Work. Barcelona, CA, Spain, 2014-2015

MW code groupings were observed according to the WC valuation levels. As shown in Figure 2, at the level of the “Very Good” WCs corresponded the following codes of meaning: autonomy, achievement and well-being. The “Good” WCs were associated with satisfaction, opportunities, good social relations, vigor, effectiveness, good socioeconomic conditions, commitment, responsibility, competence, ethics, care, economic recognition and general recognition. The “Bad” WCs corresponded to the elements of exhaustion, inappropriate work, negative climate, disorganization, overload, poor socioeconomic conditions, little recognition, malaise, injustice, organizational cynicism, dissatisfaction, mismanagement, scarce resources and inefficacy. Finally, the level of “Very Bad” WCs corresponded with depersonalization. 

In Table 2, this distribution of the ST codes is reflected according to the levels of assessment of the CT. 

**Tabela 2:** Distribuição dos códigos de Significados do Trabalho (ST) segundo níveis de avaliação das Condições de Trabalho (NACT). Barcelona, CA, Espanha, 2014-2015.

Work Meaning Codes	Levels of valuation of working conditions (LVWC*)													
	Very Bad			Bad			Good			Very Good			Accumulated	
	n^†^	%		n^†^	%		n^†^	%		n^†^	%		N^‡^	%
Overload	19	4.2		148	32.4		260	56.9		30	6.6		457	5.7
Bad management	10	8.9		63	55.8		40	35.4		0	0.0		113	1.4
Disorganization Tasks	3	3.2		49	52.7		40	43.0		1	1.1		93	1.2
Negative Climate	9	3.6		124	50.0		108	43.5		7	2.8		248	3.1
Injustice	6	5.2		69	60.0		40	34.8		0	0.0		115	1.4
Inappropriate work	4	3.2		56	45.2		56	45.2		8	6.5		124	1.5
Lack of Resources	3	9.4		13	40.6		15	46.9		1	3.1		32	0.4
Poor socioeconomic conditions	30	5.9		188	36.9		275	54.0		16	3.1		509	6.3
Dissatisfaction	11	7.9		80	57.6		47	33.8		1	0.7		139	1.7
Discomfort	40	8.3		237	49.3		197	41.0		7	1.5		481	6.0
Exhaustion	20	3.6		223	39.8		296	52.9		21	3.8		560	7.0
Organizational Cynicism	7	6.7		62	59.0		34	32.4		2	1.9		105	1.3
Depersonalization	7	17.5		22	55.0		11	27.5		0	0.0		40	0.5
Inefficacy	1	2.1		35	74.5		10	21.3		1	2.1		47	0.6
Good socioeconomic Conditions	2	1.3		26	17.0		95	62.1		30	19.6		153	1.9
Opportunities	2	0.6		48	14.0		228	66.7		64	18.7		342	4.3
Satisfaction	3	1.1		43	15.4		172	61.4		62	22.1		280	3.5
Wellbeing	2	0.5		49	12.5		256	65.3		85	21.7		392	4.9
Vigor	2	0.7		49	16.6		182	61.7		62	21.0		295	3.7
Commitment	3	0.7		63	14.9		273	64.7		83	19.7		422	5.2
Ethics	1	0.7		28	20.3		92	66.7		17	12.3		138	1.7
Good social Relations	3	0.7		77	17.3		293	66.0		71	16.0		444	5.5
Realization	0	0.0		10	8.9		79	70.5		23	20.5		112	1.4
Efficacy	5	0.9		83	15.1		369	67.0		94	17.1		551	6.9
Competences	2	2.7		16	21.6		42	56.8		14	18.9		74	0.9
Care	21	1.9		222	20.5		692	63.8		149	13.7		1084	13.5
Responsibility	2	0.6		66	18.3		242	67.0		51	14.1		361	4.5
Autonomy	0	0.0		10	10.1		62	62.6		27	27.3		99	1.2
Little Recognition	2	1.8		61	54.0		49	43.4		1	0.9		113	1.4
Recognition	0	0.0		16	27.6		30	51.7		12	20.7		58	0.7
Economic Recognition	1	1.6		16	25.8		39	62.9		6	9.7		62	0.8
Frequencies (n) and Percentages (%)	221	2.7		2252	28.0		4624	57.5		946	11.8		8043	100

*LVWC: Levels of valuation of Working Conditions

† n: frequency of meaning of work codes according to levels of assessment of working conditions;

‡ N: accumulated frequency by codes of meaning of work.

## Discussion

The results of this research are in general agreement with the information provided by the literature on the work experience and quality of working life for nursing professionals in the new environment resulting from the successive contemporary reforms of the health system. However, the results include some content that points beyond what is already known. First, the results allow for synthesizing, condensing and qualifying the effects of the political, managerial and technological innovations observed in the most diverse health contexts. Second, the results show how these new organizational demands impact nurses psychologically and how they are approached psychologically in a doubly critical scenario: a reform of health services accelerated and intensified by strong financial constraints, which translates into strategies to cut staff and increase the ratio of patients per professional, thereby increasing pressure on health care by a quantitative and qualitative over demand of care and cognitive and emotional work.

As a whole, the information provided by the study revolves around three main axes. First, the participants show a high coherence between their numerical assessment of their own working conditions and their textual characterization of the meanings of their work experience. Second, they think of their profession in terms of a caring relationship and perceive their work and professional experience in relatively positive terms, indicating aspects to be protected and strengthened. Third, they also report important shortcomings, deficiencies and dysfunctions in the design, organization and management of this work and the conditions of that professional exercise. This negative aspect is especially marked by work overload and concepts associated with it, such as the lack of time and human resources available to deal effectively with organizational demands. Other highlights are a perceived lack of autonomy in work as well as opportunities for professional development.

The moderately positive assessment of the conditions for the realization of one’s own work experience is in line with what has been gathered by current reports on the quality of work life within the general context of contemporary working conditions^17^ and also on what specifically concerns the provision of public services to persons^26^ ^-^ ^27^ and, within this group, those of the health sector in particular^1^ ^,^ ^4^ ^,^ ^21^ ^-^ ^23^ and specifically those in the field of nursing^(2- 3,13,18,28-31)^. The vision provided by the participants is also consistent with what the literature reports regarding the dark side of the characteristics and tendencies of working conditions in general, both for their psychosocial risk factors^16^ ^-^ ^18^ and because of the lack of wellbeing in health care work^5^ ^-^ ^7^ ^,^ ^10^ ^-^ ^12^ ^,^ ^26^ ^-^ ^27^ ^,^ ^30^ ^-^ ^31^.

While the numbers presented indicate the relative levels of satisfaction for the conditions of nursing work in the social and organizational environment studied, the codes of meaning associated with them give names to the aspects of those conditions that need to be strengthened and those that need to be modified.

Overall, the study contains some notable weaknesses and strengths. Among its methodological limitations is the use of self-report measures within the framework of a cross-sectional design, which in turn did not allow for causal inferences. However, none of these characteristics hampered the achievement of the research goal concerning the relationship between numerical scores and verbal responses referring to the same phenomena of experience of working conditions. However, the fact that fieldwork was developed in the context of a crisis in the Spanish health system’s financing, which had a significant impact on working conditions in nursing in Barcelona, ​​raises reasonable doubts as to whether the results presented would have been different if they had been obtained before or after this critical period. Among the strengths of the research, we highlight the breadth of the thematic field covered and its mixed design, which combined the use of numerous scales composed of a series of closed items for the collection of quantitative data, with an open question as a source of the qualitative data. This allowed the implementation of various techniques for studying the information obtained, such as statistical analysis, thematic content and correspondences. The agenda for future studies linked to the present includes the challenge of improving understanding of the mechanisms involved in an observed paradox: the nursing professionals who participated in the research were generally highly affected by a life care practice experienced as very stressful, exhausting and fatiguing while they maintained a positive vision of it, a high professional self-esteem and a strong involvement in the work.

## Conclusion

The eight thousand key words with which the respondents summarized their work experience in their current organizational context agreed with their numerical assessments of the conditions of their professional practice, giving such scores a more precise meaning. In this respect, the contribution of this study has a twofold aspect: on the one hand, it ratifies in the current Barcelona environment quantitative aspects already known regarding the real conditions of the contemporary practice of health care. On the other, it involves progress in and deepening of the qualitative knowledge of the investigated reality by specifying the positive and negative nuances of current nursing work experience with words, categories and semantic codes.

By showing the image of an ambivalent work experience characterized by the tension between the vocation to care and the imposition of a chronic task overload, professionals have noted challenges for reflection and paths for policies committed to a double mission: strengthening the positive aspects and correcting the negative aspects of the nursing staff’s work conditions, with a view to improving their occupational health and well-being while optimizing the quality of the service they provide to their patients and their community.
